# Circular RNA MYLK as a prognostic biomarker in patients with cancers: A systematic review and meta‐analysis

**DOI:** 10.1002/cnr2.1653

**Published:** 2022-06-14

**Authors:** Roham Foroumadi, Sina Rashedi, Sara Asgarian, Mahta Mardani, Mohammad Keykhaei, Hossein Farrokhpour, Salar Javanshir, Rojin Sarallah, Nima Rezaei

**Affiliations:** ^1^ School of Medicine Tehran University of Medical Sciences Tehran Iran; ^2^ Network of Immunity in Infection, Malignancy and Autoimmunity (NIIMA) Universal Scientific Education and Research Network (USERN) Tehran Iran; ^3^ Non‐Communicable Diseases Research Center Endocrinology and Metabolism Population Sciences Institute, Tehran University of Medical Sciences Tehran Iran; ^4^ School of Medicine Iran University of Medical Sciences Tehran Iran; ^5^ School of Medicine Tehran Medical Sciences Branch, Islamic Azad University Tehran Iran; ^6^ Research Center for Immunodeficiencies Children's Medical Center, Tehran University of Medical Sciences Tehran Iran; ^7^ Department of Immunology School of Medicine, Tehran University of Medical Sciences Tehran Iran

**Keywords:** circular RNA, meta‐analysis, microRNA, MYLK, neoplasm, prognosis

## Abstract

**Background:**

Circular RNA (circRNA) myosin light chain kinase (circMYLK) has recently received increasing attention in cancer biology. Several studies have suggested that circMYLK expression is linked to prognosis and clinicopathological characteristics of various malignancies.

**Aims:**

This study was carried out to systematically review the impact of circMYLK on the progression of multiple cancers and assess the significance of circMYLK in the prognosis and clinicopathological features of the patients.

**Methods:**

PubMed, Web of Science, and Embase were systematically searched until July 2, 2021. For qualitative synthesis, the signaling pathways of circMYLK in the progression of different cancers were summarized. Regarding the meta‐analysis, overall survival (OS) and eight clinicopathological characteristics of patients with cancers were addressed. Odds ratios (ORs) and hazard ratios (HRs) were calculated to assess the association of circMYLK with prognostic and clinicopathological features.

**Results:**

Twelve studies investigating the role of circMYLK in cancer progression met the inclusion criteria. Among these, seven studies investigated the prognostic significance of circMYLK, and nine studies ascertained the clinicopathological importance of circMYLK in patients with various malignancies. CircMYLK acts as a tumor promoter circRNA, leading to migration, proliferation, invasion, and metastasis of neoplastic cells and inhibiting their apoptosis through interaction with several miRNAs and corresponding downstream signaling pathways. Overexpression of circMYLK was correlated with poor OS (HR = 1.75; 95% confidence interval [CI] 1.52–2.02) and larger tumor size (OR = 2.90; 95% CI 1.03–8.15), higher T stage (OR = 2.49; 95% CI 1.20–5.18), lymph node metastasis (OR = 2.55; 95% CI 1.41–4.62), and higher TNM stage (OR = 4.62; 95% CI 2.99–7.14).

**Conclusions:**

CircMYLK is involved in the progression of numerous cancers via different signaling pathways. This circRNA can serve as a promising prognostic biomarker for several types of malignancies. Furthermore, high expression of circMYLK is associated with advanced clinicopathological characteristics in various tumors.

## 1 INTRODUCTION

Cancer, a growing health and economic issue, is one of the leading causes of mortality and morbidity worldwide.^1^ Despite recent advances in multiple cancer fields, including diagnosis, prognosis assessment, and treatment, the overall cancer‐related mortality rate is estimated to continue to rise. Cancer's rising importance is due to the increase in the number of newly‐diagnosed cases and inadequate understanding of the molecular mechanisms underlying cancer promotion.^2^ The development of novel molecular biomarkers brings about a better diagnostic and prognostic evaluation of oncologic patients. Additionally, the potential role of these biomarkers as therapeutic targets holds great clinical implications, possibly contributing to advances in cancer treatment strategies.

Circular RNAs (circRNAs) are closed‐loop long non‐coding RNAs with covalently connected 5′ and 3′ termini formed by back‐splicing within genes.^3^ CircRNAs are widely expressed and conserved evolutionary in mammalian cells with tissue‐specific expression patterns.^4^ CircRNAs regulate cellular physiology through various molecular pathways, including acting as sponges for microRNAs (miRNA or miR) or RNA‐binding proteins to alter the gene expression or regulation of protein translation.^4^ Previous investigations have revealed the pivotal role of circRNAs in many complex functions and mechanisms, including the proliferation, metastasis, and invasion of various malignancies, for example, breast cancer and colon cancer.^5^ Moreover, a growing body of evidence has identified the beneficial roles of circRNAs as diagnostic and prognostic biomarkers in various malignancies.^5^

Recently, circRNA myosin light chain kinase (circMYLK) has received increasing attention in cancer biology and has been studied in several malignancies.^6^ Based on the CircBase database annotation, circMYLK is spliced from the MYLK gene on chr3:123471177–123 512 691 and has a length of 376 nt.^7^ Current investigations have found the oncogenic role of circMYLK, which is overexpressed in several cancer types, including bladder cancer,^7^, ^8^ non‐small‐cell lung cancer (NSCLC),^9^ and hepatocellular carcinoma (HCC).^10^, ^11^ This circular transcript participates in cancer‐related critical pathways, such as VEGFA/VEGFR2, MEK/ERK, and NF‐κB pathways.^7^, ^12^ Furthermore, several studies have suggested that circMYLK expression is associated with the various malignancies' prognosis and clinicopathological characteristics.^7^, ^8^, ^9^, ^10^, ^11^, ^13^, ^14^, ^15^, ^16^ Despite these promising results, no prior studies have comprehensively reviewed the relationship between circMYLK expression and clinical outcomes of cancer patients. Herein, we carried out a systematic review and meta‐analysis to determine the role of circMYLK in cancer progression and provide a precise predictive value of circMYLK in the prognosis and clinicopathological features of patients with various cancers.

## 2 MATERIALS AND METHODS

The Preferred Reporting Items for Systematic Reviews and Meta‐Analyses (PRISMA) statement was used to conduct and report this systematic review and meta‐analysis.^17^ Given the acquisition of ethical and Institutional Review Board (IRB) approval for each of the included articles, no approvals were required for the present report.

### 2.1 Search strategy

Three electronic databases (Web of Science, Embase, and PubMed) were systematically searched using the keywords [“RNA, Circular”] AND [“MYLK” OR “circMYLK”] until July 2, 2021, without any language or study type restrictions (Additional file 1: Search strategy). Furthermore, a manual search was conducted within the references of included studies and review articles to find further records.

### 2.2 Eligibility criteria and study selection

After removing the duplicate records, two investigators independently evaluated the titles and abstracts of the studies to obtain those assessing the significance of circMYLK in cancers. Studies without any description of circMYLK in malignancies were excluded. At this stage, the full texts of the remaining articles were reviewed for inclusion by the same investigators based on the following eligibility criteria:

- The patient population of the study consisted of adult (age ≥ 18 years) patients diagnosed with any type of cancer.

AND

II The association between the expression of circMYLK and cancer progression was assessed.

The non‐original studies and studies with a solely bioinformatic approach were excluded. A third investigator double‐checked the process of study selection.

### 2.3 Data extraction and quality assessment

The data regarding the basic study characteristics (first author, country, and year), cancer type, detection sample and method of detection, the expression pattern of circMYLK, and its downstream signaling pathway were extracted from all the included studies for qualitative synthesis of the results.

For the meta‐analysis, overall survival (OS) was regarded as the primary prognostic outcome, and hazard ratios (HRs) and their corresponding 95% confidence intervals (CIs) were estimated based on the provided data and Kaplan–Meier survival curves, according to the methods described previously.^18^ The risk of bias within the studies included in the prognostic analysis was assessed using the Newcastle‐Ottawa scale (NOS), which involves three main domains (comparability, outcome assessment, and population selection) with a maximum of nine scores.^19^

Concerning the clinicopathological features, eight parameters were assumed: 1. Age (older vs. younger); 2. Gender (male vs. female); 3. Tumor size (larger vs. smaller); 4. Tumor grade (III + IV vs. I + II); 5. T stage (III + IV vs. I + II); 6. Lymph node metastasis (yes vs. no); 7. Distant metastasis (yes vs. no); and 8. TNM stage (III + IV vs. I + II). For calculation of odds ratios (ORs) and 95% CIs, the number of patients in high‐ and low‐expression groups of the circMYLK for these endpoints and *p*‐values comparing these groups were collected.

### 2.4 Statistical analysis

For the prognostic meta‐analysis, pooled HRs and 95% CIs were calculated to explore the association between circMYLK expression and the OS of oncologic patients. Sensitivity analysis was conducted to assess each study's contribution to the pooled HRs by excluding one study at a time from the meta‐analysis. Pooled ORs and 95% CIs were calculated for each endpoint regarding clinicopathological features. Forest plots were constructed for OS and clinicopathological features of patients with cancers. The statistical heterogeneity was explored by Cochrane's Q test (*p*‐value <0.05 signifying heterogeneity) and Higgins' I‐squared test (I^2^ > 50% indicating heterogeneity).^20^ In case of high statistical heterogeneity (I^2^ > 50%), random‐effect models were employed to pool effect sizes; otherwise, fixed‐effect models were implemented. Publication bias was investigated by visual assessment of funnel plots and Begg's test for prognostic meta‐analysis. All the analyses were performed in Stata (version 14.2; Stata Corp, College Station, Texas, USA) and RevMan (version 5.4), with the *p*‐value <0.05 representing statistical significance.

## 3 RESULTS

### 3.1 Search results and study characteristics

**FIGURE 1:**
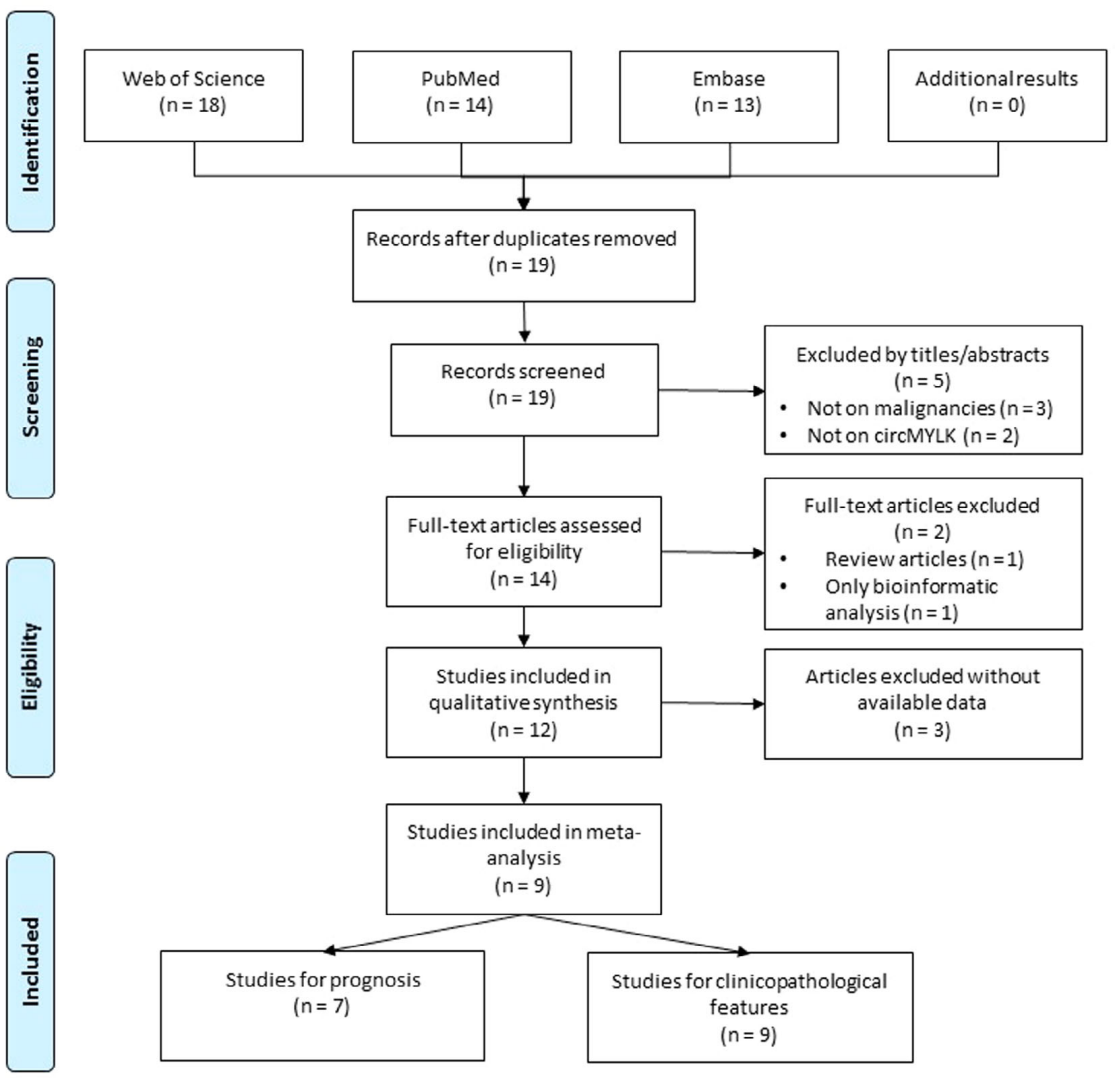
Flow diagram of study selection

The search within electronic databases yielded 19 distinct articles, and no additional studies were identified by manual search. Five studies were excluded by title and abstract review. Moreover, one review article and one investigation with only bioinformatic analysis were excluded after full‐text reviews (Figure 1). Ultimately, 12 studies were included in the qualitative synthesis of this review^7^, ^8^, ^9^, ^10^, ^11^, ^12^, ^13^, ^14^, ^15^, ^16^, ^21^, ^22^ (Table 1); however, three studies did not provide data regarding prognostic or clinicopathological features^12^, ^21^, ^22^; therefore, nine articles were included in the meta‐analysis. Seven of these studies investigated the prognostic significance of circMYLK, and nine studies ascertained the clinicopathological importance of circMYLK in patients with malignancies.

**TABLE 1:** Characteristics of the included studies investigating circMYLK in cancers.

Cancer type	Expression level	Detection method	Target miRNA or proteins	Downstream proteins and signaling pathways	Function/clinical associations	Model	Sample/cell line	Country	References
Bladder cancer	Upregulated	qRT‐PCR	miR‐29a	VEGFA/VEGFR2 and Ras/ERK signaling pathway	Promotes the growth, proliferation, migration, angiogenesis, metastasis, and epithelial‐mesenchymal transition	In vitro, in vivo, mice, human	EJ, T24, 5673 and BIU‐87/4‐week‐old male BALB/c mice/32 bladder carcinomas and matched para‐carcinoma tissues	China	Zhong, Z. 2017
Prostate cancer	Upregulated	qRT‐PCR	miR‐29a	Targets miR‐29a	Promotes proliferation, invasion, migration; Inhibits apoptosis	In vitro, human	DU145, LNCaP, PC‐3, and PC‐3MIE8/17 paired cancer and matched non‐tumorous tissues	China	Dai, Y. 2018
Laryngeal SCC	Upregulated	qRT‐PCR	miR‐195, cyclin D1	miR‐195/cyclin D1 axis	Promotes cell proliferation & G1/S cell cycle transition; Arrests AMC‐HN8 cells in G0/G1 phase	In vitro, human	AMC‐HN8, Tu‐177, human bronchial epithelial cell line (16 HBE)/72 cancer & adjacent non‐tumorous tissues	China	Duan, Z. 2019
HCC	Upregulated	qRT‐PCR	miR‐362‐3p, rab‐23	miR‐362‐3p/rab‐23 axis	Promotes proliferation, invasion, intra‐ and extrahepatic metastasis, and tumor size; Facilitates cancer progression	In vitro, in vivo, human	Huh7, Hep3B, HCCLM3, SK‐Hep1, PLC, HepG2 cell lines/mice/62 cancer tissues and adjacent non‐tumorous tissues	China	Li, Z. 2019
Cervical cancer	Upregulated	qRT‐PCR	miR‐1301‐3p, RHEB,	RHEB‐dependent mTOR pathway	Promotes cell growth, cell proliferation, viability; Inhibits apoptosis	In vitro	DoTc2 4510, HCC94, C‐33A, HT3	China	Chen, R. 2020
Laryngocarcinoma	Upregulated	qRT‐PCR	miR‐145‐5p	MEK/ERK and NF‐κB cascades	Promotes viability, invasion, migration; Inhibits apoptosis	In vitro	Hep‐2	China	Chen, Y. 2020
HCC	Upregulated	qRT‐PCR	miR‐29a, KMT5C	miR‐29a/KMT5C signaling pathway	Promotes cell invasion, proliferation, and migration; Inhibits apoptosis	In vitro, in vivo, human	MHCC‐97H; HCC‐LM3; nude mouse/60 HCC versus adjacent non‐tumorous tissues	China	Gao, J. 2020
Renal cancer	Upregulated	qRT‐PCR	miR‐513a‐5p, VEGFC	miR‐513a‐5p/VEGFC signaling pathway	Promotes tumor growth, cell proliferation, distance metastasis, and poor prognosis	In vitro, in vivo, human	HK‐2 cell, ACHN, 786‐O, Caki‐2, and ten nude mice/71 cancer tissue & matched non‐tumorous tissue samples	China	Li, J. 2020
NSCLC	Upregulated	qRT‐PCR	miR‐195‐5p	miR‐195‐5p/GLUT3 regulatory network	Associates with deleterious clinicopathological characteristics and poor prognosis; Promotes proliferation, colony formation, migration, invasion, glycolysis, and lactate production	In vitro, in vivo, human	16HBE, H23, A549, H1299, and SPC‐A1/Non‐small cell lung cancer tissues and matched adjacent normal tissues	China	Xiong, S. 2020
Ovarian cancer	Upregulated	qRT‐PCR	miR‐652	Targets miR‐652	Promotes proliferation and malignant progression of ovarian cancer; Correlates with pathological staging, poor prognosis, and lower overall survival	In vitro, in vivo, human	Human ovarian cancer cells (SKOV3, OVCAR3, PEO1, 3AO, A2780, CAOV3) and normal human ovarian surface epithelial cells/46 tumor tissue samples and corresponding adjacent normal tissues	China	Zhao, Y. 2020
Colorectal cancer	Upregulated	qRT‐PCR	NA	NA	Promotes proliferation, invasion, migration; Elevated tumor size, upregulated TNM stage, lymph node metastasis, and distant metastasis; Inhibits apoptosis; Correlates with poor prognosis (poor overall survival and progression‐free survival)	In vitro, in vivo, human	Colorectal cancer cell lines (HCT116, SW480, SW620, HT29, and LOVO) and a normal intestinal epithelial cell line (NCM460)/90 cancer tissues and adjacent normal para‐cancerous tissues	China	Huang, L. 2021
Bladder cancer	Upregulated	qRT‐PCR	miR‐34a	miR‐34a/CCND3 regulatory network	Promotes cell invasion, proliferation, and migration; Inhibits apoptosis	In vitro, in vivo, human	SW780, T24, J82, and RT4, and bladder epithelium cells non‐cancer, SV‐HUC‐1/50 bladder cancer tissues and matched adjacent normal tissues	China	Ye, W. 2021

Abbreviations: HCC, hepatocellular carcinoma; miRNA, microRNA; NA, not available; NSCLC, non‐small cell lung cancer; qRT‐PCR, quantitative real‐time polymerase chain reaction; SCC, squamous cell carcinoma.

Table 1 summarizes the characteristics of included studies. All studies were from China and published between 2017 and 2021. Using the quantitative real‐time polymerase chain reaction (qRT‐PCR), the studies revealed upregulation of circMYLK in different malignant tissue samples, including bladder cancer,^7^, ^8^ HCC,^10^, ^11^ laryngeal cancer,^12^, ^13^ NSCLC,^9^ renal cancer,^14^ ovarian cancer,^15^ prostate cancer,^22^ cervical cancer,^21^ and colorectal cancer.^16^ According to these studies, circMYLK acts as a tumor promoter circRNA leading to migration, invasion, proliferation, and metastasis of neoplastic cells and inhibiting their apoptosis through interaction with several miRNAs and corresponding downstream signaling pathways.

### 3.2 Prognosis

**FIGURE 2:**
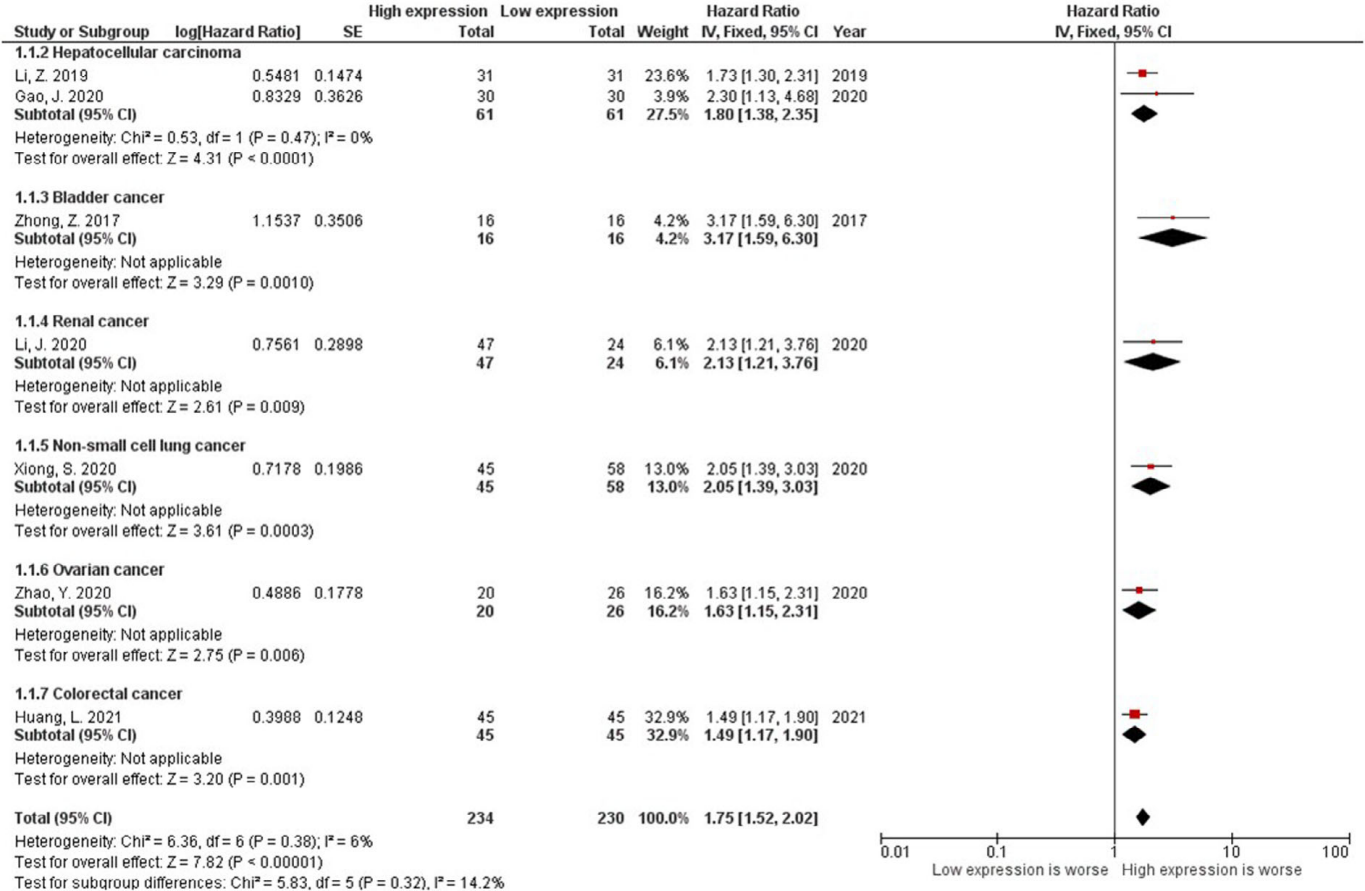
Forest plot of circMYLK for overall survival (OS) of patients with cancers.

Concerning the prognostic endpoint, seven studies consisting of 464 patients addressed the OS of patients with six distinct malignancies (two studies for HCC and one study for renal cancer, bladder cancer, NSCLC, ovarian cancer, and colorectal cancer) with the maximum follow‐up duration ranging from 30 to 100 months (Table 2).^7^, ^9^, ^10^, ^11^, ^14^, ^15^, ^16^ Based on NOS, the methodological quality of included studies was six to seven out of nine scores, mainly lacking the scores for adjustment factors (Figure S1). All studies determined upregulation of circMYLK to be associated with poor OS of patients with the mentioned cancers, with the pooled HR = 1.75 [(95% CI 1.52–2.02); P < 0.01] (Figure 2). No statistically significant heterogeneity was detected in this meta‐analysis (I^2^ = 6%, P_Q_ = 0.38). No evidence of publication bias was observed utilizing funnel plot assessment and Begg's test (*p* = 0.07) (Figure S2). Sensitivity analysis showed that the results were consistent after omitting each study from the pooled analysis (Figure S3).

**TABLE 2:** Characteristics of the studies included in the prognostic analysis.

Study, year	Cancer type	Country	Detection method	Detected sample	Expression	Case number		Cut‐off	Outcomes	Maximum follow‐up (months)	NOS score
						High level	Low level				
Zhong, Z. 2017	Bladder cancer	China	qRT‐PCR	Tissue	Upregulated	16	16	Median	OS	30	6
Li, Z. 2019	HCC	China	qRT‐PCR	Tissue	Upregulated	31	31	Median	OS	60	7
Gao, J. 2020	HCC	China	qRT‐PCR	Tissue	Upregulated	30	30	Median	OS	60	7
Li, J. 2020	Renal cancer	China	qRT‐PCR	Tissue	Upregulated	49	22	Relative expression	OS	60	7
Xiong, S. 2020	NSCLC	China	qRT‐PCR	Tissue	Upregulated	45	58	Relative expression	OS	60	7
Zhao, Y. 2020	Ovarian cancer	China	qRT‐PCR	Tissue	Upregulated	20	26	Relative expression	OS	70	7
Huang, L. 2021	Colorectal cancer	China	qRT‐PCR	Tissue	Upregulated	45	45	Median	OS, PFS	100	7

Abbreviations: HCC, hepatocellular carcinoma; NOS, Newcastle‐Ottawa scale; NSCLC, non‐small cell lung cancer; OS, overall survival; PFS, progression‐free survival; qRT‐PCR, quantitative real‐time polymerase chain reaction.

### 3.3 Clinicopathological features

**FIGURE 3:**
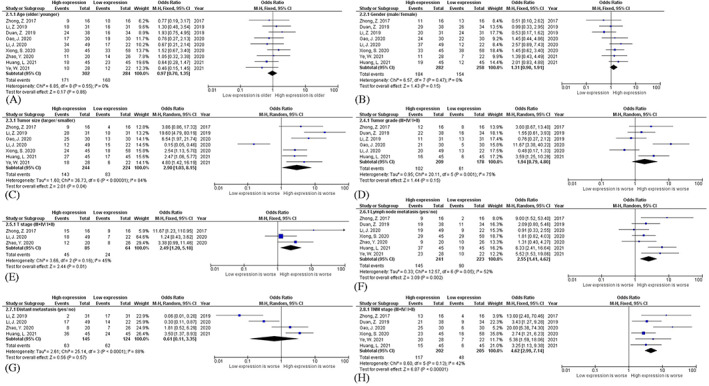
Forest plots of circMYLK for clinicopathological features of patients with cancers, including (A) Age (older vs. younger); (B) Gender (male vs. female); (C) Tumor size (larger vs. smaller); (D) Tumor grade (III + IV vs. I + II); (E) T stage (III + IV vs. I + II); (F) Lymph node metastasis (yes vs. no); (G) Distant metastasis (yes vs. no); and (H) TNM stage (III + IV vs. I + II).

For the clinicopathological characteristics, nine studies, encompassing 586 patients were included (Table 3).^7^, ^8^, ^9^, ^10^, ^11^, ^13^, ^14^, ^15^, ^16^ The overexpression of circMYLK was correlated with larger tumor size [OR = 2.90 (95% CI 1.03–8.15); *p* = 0.04; heterogeneity statistics: I^2^ = 84%, P_Q_ <0.01], higher T stage [OR = 2.49 (95% CI 1.20–5.18); *p* = 0.01; heterogeneity statistics: I^2^ = 45%, P_Q_ = 0.16], lymph node metastasis [OR = 2.55 (95% CI 1.41–4.62); P < 0.01; heterogeneity statistics: I^2^ = 52%, P_Q_ = 0.05], and higher TNM stage [OR = 4.62 (95% CI 2.99–7.14); P < 0.01; heterogeneity statistics: I^2^ = 42%, P_Q_ = 0.13] (Figure 3). The expression of circMYLK was not associated with age [OR = 0.97 (95% CI 0.70–1.35); *p* = 0.86; heterogeneity statistics: I^2^ = 0%, P_Q_ = 0.55], gender [OR = 1.31 (95% CI 0.90–1.91); *p* = 0.15; heterogeneity statistics: I^2^ = 0%, P_Q_ = 0.47], tumor grade [OR = 1.94 (95% CI 0.79–4.80); *p* = 0.15; heterogeneity statistics: I^2^ = 75%, P_Q_ <0.01], and distant metastasis [OR = 0.61 (95% CI 0.11–3.35); *p* = 0.57; heterogeneity statistics: I^2^ = 88%, P_Q_ <0.01] (Figure 3).

**TABLE 3:** Characteristics of the studies regarding the role of circMYLK and clinicopathological features of cancers.

Study, year	Cancer type	Country	Detection method	Detected sample	Expression	Case number		Age (older/younger)	Gender (male/female)	Tumor size (larger/smaller)	Tumor grade (III + IV/I + II)	T stage (III + IV/I + II)	Lymph node metastasis (yes/no)	Distant metastasis (yes/no)	TNM stage (III + IV/I + II)
						High level	Low level								
Zhong, Z. 2017	Bladder cancer	China	qRT‐PCR	Tissue	Upregulated	16	16	1.000	0.685	0.148	0.273	0.037	0.023	NA	0.003
Duan, Z. 2019	Laryngeal SCC	China	qRT‐PCR	Tissue	Upregulated	38	34	0.170	0.988	NA	0.358	NA	0.129	NA	0.013
Li, Z. 2019	HCC	China	qRT‐PCR	Tissue	Upregulated	31	31	0.515	0.302	0.003	0.173	NA	NA	0.004	NA
Gao, J. 2020	HCC	China	qRT‐PCR	Tissue	Upregulated	30	30	0.635	0.561	0.002	<0.001	NA	NA	NA	0.003
Li, J. 2020	Renal cancer	China	qRT‐PCR	Tissue	Upregulated	47	24	0.787	0.077	0.001	0.240	0.560	0.971	0.043	NA
Xiong, S. 2020	NSCLC	China	qRT‐PCR	Tissue	Upregulated	45	58	0.313	0.395	0.022	NA	NA	0.143	NA	0.015
Zhao, Y. 2020	Ovarian cancer	China	qRT‐PCR	Tissue	Upregulated	20	26	0.938	NA	NA	NA	0.470	0.655	0.348	NA
Huang, L. 2021	Colorectal cancer	China	qRT‐PCR	Tissue	Upregulated	45	45	0.290	0.120	0.035	0.011	NA	<0.001	0.007	0.036
Ye, W. 2021	Bladder cancer	China	qRT‐PCR	Tissue	Upregulated	28	22	0.183	0.585	0.009	NA	NA	0.006	NA	0.005

*Note*: The numbers represent the *p*‐values for the association between the circMYLK and clinicopathological features of malignancies in each study.

Abbreviations: HCC, hepatocellular carcinoma; NA, not available; NSCLC, non‐small cell lung cancer; qRT‐PCR, quantitative real‐time polymerase chain reaction; SCC, squamous cell carcinoma.

## 4 DISCUSSION

Despite recent advancements in treatment options, cancer remains one of the world's top causes of death. According to the global cancer burden, it is anticipated that 22.2 million additional cancer cases will be diagnosed in 184 countries by 2030.^23^ This surge in patients with cancer necessitates the development of new biologically specific biomarkers for early cancer detection. Aside from cancer diagnosis, biomarkers are a critical consideration that may influence clinical decision‐making and determine disease course.

Recent investigations indicated that circRNAs are involved in various tumor‐related biological pathways and are emerging as potential biomarkers for cancer diagnosis and prognosis.^24^ For instance, Huang et al. indicated that circRNAs could be important biomarkers for the diagnosis and prognosis of HCC.^25^ According to Yang et al., circRNAs can serve as prognostic markers in lung cancer.^26^ As shown by Li et al., abnormally expressed circRNAs have demonstrated promising potential as diagnostic biomarkers in colorectal cancer and prognostic factors to determine the overall survival in these populations.^27^ Furthermore, Wang et al. proposed that certain circRNAs are associated with the prognosis and clinicopathological features of bladder cancer patients.^28^ As discussed in this study, circMYLK regulates pivotal signaling pathways and cancer‐related cellular processes, including cell proliferation, invasion, and apoptosis through miRNA modulation. Moreover, circMYLK can be a valuable prognostic factor in various cancers, including HCC, renal cancer, bladder cancer, NSCLC, ovarian cancer, and colorectal cancer.^16^

Many studies have investigated the role of a single circRNA in multiple cancers. Sun et al. confirmed that overexpression of circ‐ITCH is associated with poor clinicopathological parameters in several cancer types.^29^ CircRNA CDR1as, according to Zou et al., is a reliable prognostic and diagnostic biomarker in solid tumors.^30^ Furthermore, Lin et al. proposed that high circPVT1 expression is correlated with poor prognosis in malignancies.^31^ Chao et al. discovered that circSMARCA5 is a promising biomarker in human cancers, which may assist in the management of cancer patients in the future.^32^ Exploring the potential of circRNAs as biomarkers can provide valuable information for clinical decision‐making. However, the effect of circMYLK on the prognosis of cancers has not been systematically analyzed.

**FIGURE 4:**
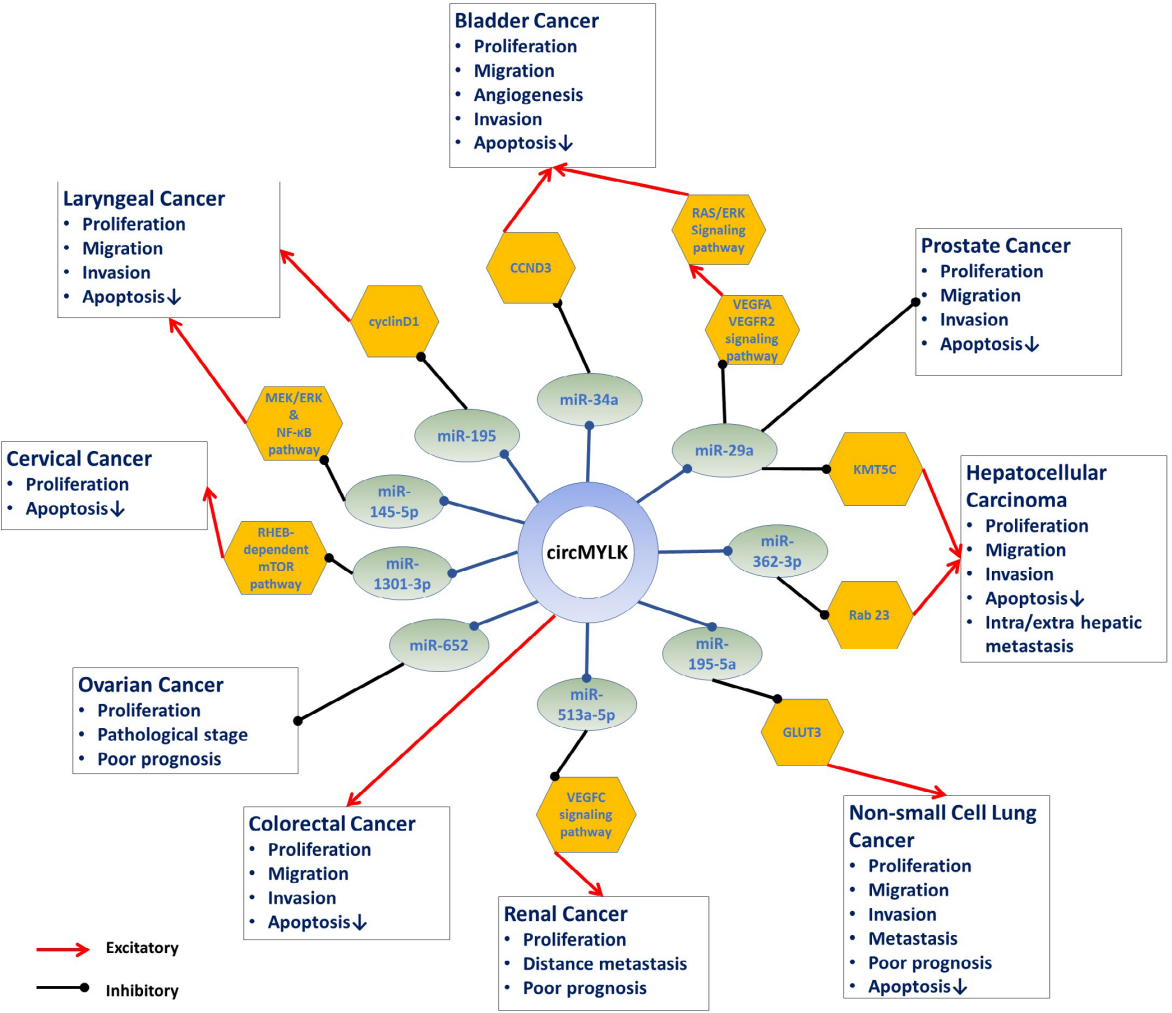
Schematic diagram of circMYLK's mechanism in cancers

This article is the first systematic review and meta‐analysis regarding the pathophysiological and clinical significance of circMYLK in cancers. Based on these studies, elevated circMYLK expression was correlated with a poor OS and worse clinicopathological features, such as higher TNM stage, larger tumor size, and lymph node metastasis, which are all hallmarks of advanced tumors. We summarized the identified modes of action and the involved pathways in circMYLK‐regulated tumor progression in Figure 4.

CircMYLK can act as a competing endogenous RNA for multiple miRNAs and promote cancer progression by sponging these miRNAs. The downregulation of miR‐29a contributes to cancer progression by stimulating VEGFA/VEGFR2 and the downstream Ras/ERK signaling pathway, leading to increased angiogenic properties and metastatic features.^6^, ^22^ CircMYLK accelerates prostate cancer, bladder cancer, and HCC progression via mediating miR‐29a. In addition, also by targeting miR‐34a, circMYLK may contribute to bladder cancer progression.^8^ In another study, circMYLK promoted HCC progression by sponging miR‐362‐3p, causing Rab23 upregulation.^10^ By interacting with miR‐652, circMYLK promotes the development of ovarian cancer cells.^15^ Additionally, Li et al. found that circMYLK can act as a modulator of the miR‐513a‐5p/VEGFC pathway in renal cell carcinoma.^14^ CircMYLK activates RHEB, a GTPase from the Ras superfamily, via downregulation of miR‐1301‐3p in cervical cancer cells.^21^ RHEB functions as a major upstream activator of the mTOR pathway, which promotes metabolic reprogramming of glucose and glutamine in cancer cells, ensuring rapid growth and proliferation of neoplastic cells.^33^ CircMYLK can activate cyclinD1 and MEK/ERK‐NF‐κB cascades in laryngeal cancer.^12^, ^13^ MEK/ERK cascade is involved in numerous critical cellular pathways, for example, apoptosis and proliferation, through coupling cell surface receptors to transcription factors.^34^, ^35^ Besides, NF‐κB mediates cancer cell survival and proliferation alongside pathological angiogenesis by regulating the expression of numerous genes, for example, BCL2, BCLXL, and VEGF.^36^, ^37^ CircMYLK employs these oncogenic roles in laryngeal cancer by sponging miR‐195 and miR‐145‐5p.^12^, ^13^

These studies suggest that circMYLK can promote tumor proliferation and invasion through various signaling pathways. The meta‐analysis results supported the proposed pathophysiological roles of circMYLK in cancer progression, indicating the poor prognosis and advanced clinicopathological features related to upregulation of circMYLK. Further investigations are warranted to shed light on the biological pathways related to circMYLK in other malignancies.

For the first time, this systematic review and meta‐analysis comprehensively reviewed the current literature and pooled the available evidence regarding the role of circMYLK in cancers following standard procedures and employing robust statistics. However, the following limitations merit consideration. First, all of the included studies were based on the Asian (Chinese) population, which may influence our findings on a larger scale. Future studies are warranted to evaluate the role of circMYLK as a prognostic factor for patients with cancers of other races and ethnicities. Second, the HRs were not directly reported in any of the included studies. Thus, two experienced investigators estimated the HRs and 95% intervals based on the methods previously described by Tierney et al.^18^ Third, all studies addressing circMYLK in malignancies used cancer tissue specimens; thus, additional research into circMYLK in more accessible samples, such as serum, is warranted. Fourth, there was no consensus for calculating the cut‐off value for circMYLK, distinguishing high‐ and low‐expression groups. A practical cut‐off value for circMYLK should be determined to better picture the utility of circMYLK as a prognostic biomarker. Fifth, the included studies mainly address the role of circMYLK as a decoy for several miRNAs. CircRNAs are also involved in other cellular processes, resulting in cancer progression, for example, protein sequestration, functioning as protein scaffolds, or transcription regulation.^38^ Additional studies are needed to ascertain the impact of circMYLK on other cellular processes (besides miRNA sponging) related to cancer progression. Finally, the number of studies included in this meta‐analysis was limited, which can affect the statistical power of the analyses. Since the target of circMYLK may vary between various types of malignant tumors, future high‐quality multicenter large‐scale studies in different types of malignancies involving patients of various races and ethnicities are required to obtain more definitive results.

## 5 CONCLUSION

To sum up, circMYLK is involved in the progression of several cancers via different signaling pathways. This circRNA can serve as a promising prognostic biomarker for several types of malignancies, and high circMYLK expression is associated with advanced clinicopathological characteristics in various tumors. Future multicenter and high‐quality studies with patients from diverse races and ethnicities are warranted to confirm these findings.

## AUTHOR CONTRIBUTIONS

**Roham Foroumadi:** Conceptualization (equal); data curation (equal); formal analysis (equal); investigation (equal); methodology (equal); project administration (equal); writing – original draft (lead); writing – review and editing (equal). **Sina Rashedi:** Conceptualization (equal); formal analysis (equal); methodology (equal); project administration; writing – original draft (equal); writing – review and editing. **Sara Asgarian:** Data curation (equal); formal analysis (equal); writing – original draft (equal). **Mahta Mardani:** Data curation (equal); investigation (equal); writing – review and editing (equal). **Hossein Farrokhpour:** Formal analysis; methodology (equal); visualization (equal). **Salar Javanshir:** Investigation; writing – review and editing. **Rojin Sarallah:** Data curation; investigation. **Nima Rezaei:** Conceptualization (equal); project administration (lead); supervision (equal).

## FUNDING INFORMATION

This study received no specific grant from funding agencies in the commercial, public, or not‐for‐profit sectors.

## CONFLICT OF INTEREST

The authors have stated explicitly that there are no conflicts of interest in connection with this article.

## ETHICS STATEMENT

Not applicable.

## Supporting information


**Data S1.** Supporting information.

## Data Availability

The corresponding author will provide the datasets used and analyzed in this research on reasonable request.
