# Supplementary material for: Circular RNA MYLK as a prognostic biomarker in patients with cancers: A systematic review and meta‐analysis
Source: Cancer Rep (Hoboken). 2022 Jun 14;5(9):e1653. doi: 10.1002/cnr2.1653 (PMC9458501; doi:10.1002/cnr2.1653)
Supplement: Supplementary file 1 — Data S1. Supporting information. [file CNR2-5-e1653-s001.pdf]

Additional file 1

Search strategy

PubMed (July 2, 2021)

No.	Query	Results
#1	“MYLK”[All Fields] OR “circMYLK”[All Fields] OR “circ-MYLK”[All Fields] OR “hsa_circ_0002768”[All Fields] OR “has-circ-0002768”[All Fields] OR “Myosin Light Chain Kinase”[All Fields]	4,071
#2	"RNA, Circular"[Mesh] OR “circRNA*”[All Fields] OR “Closed Circular RNA*”[All Fields] OR “Circular RNA*”[All Fields] OR “Circular Intronic RNA*”[All Fields] OR “ciRNA*”[All Fields] OR “circular ribonucleic acid”[All Fields]	7,139
#3	#1 AND #2	**14**

Embase (July 2, 2021)

No.	Query	
#1	“MYLK”:ti,ab,kw OR “circMYLK”:ti,ab,kw OR “circ-MYLK”:ti,ab,kw OR “hsa_circ_0002768”:ti,ab,kw OR “has-circ-0002768”:ti,ab,kw OR “Myosin Light Chain Kinase”:ti,ab,kw	4,035
#2	"circular ribonucleic acid"/mj OR “circRNA*”:ti,ab,kw OR “Closed Circular RNA*”:ti,ab,kw OR “Circular RNA*”:ti,ab,kw OR “Circular Intronic RNA*”:ti,ab,kw OR “ciRNA*”:ti,ab,kw	7,374
#3	#1 AND #2	**13**

Web of Science (July 2, 2021)

No.	Query	Results
#1	ALL=(MYLK OR circMYLK OR circ-MYLK OR hsa_circ_0002768 OR has-circ-0002768 OR Myosin Light Chain Kinase)	7,143
#2	ALL=(circRNA* OR Closed Circular RNA* OR Circular RNA* OR Circular Intronic RNA* OR ciRNA* OR circular ribonucleic acid)	14,033
#3	#1 AND #2	**18**

Supplementary Figures

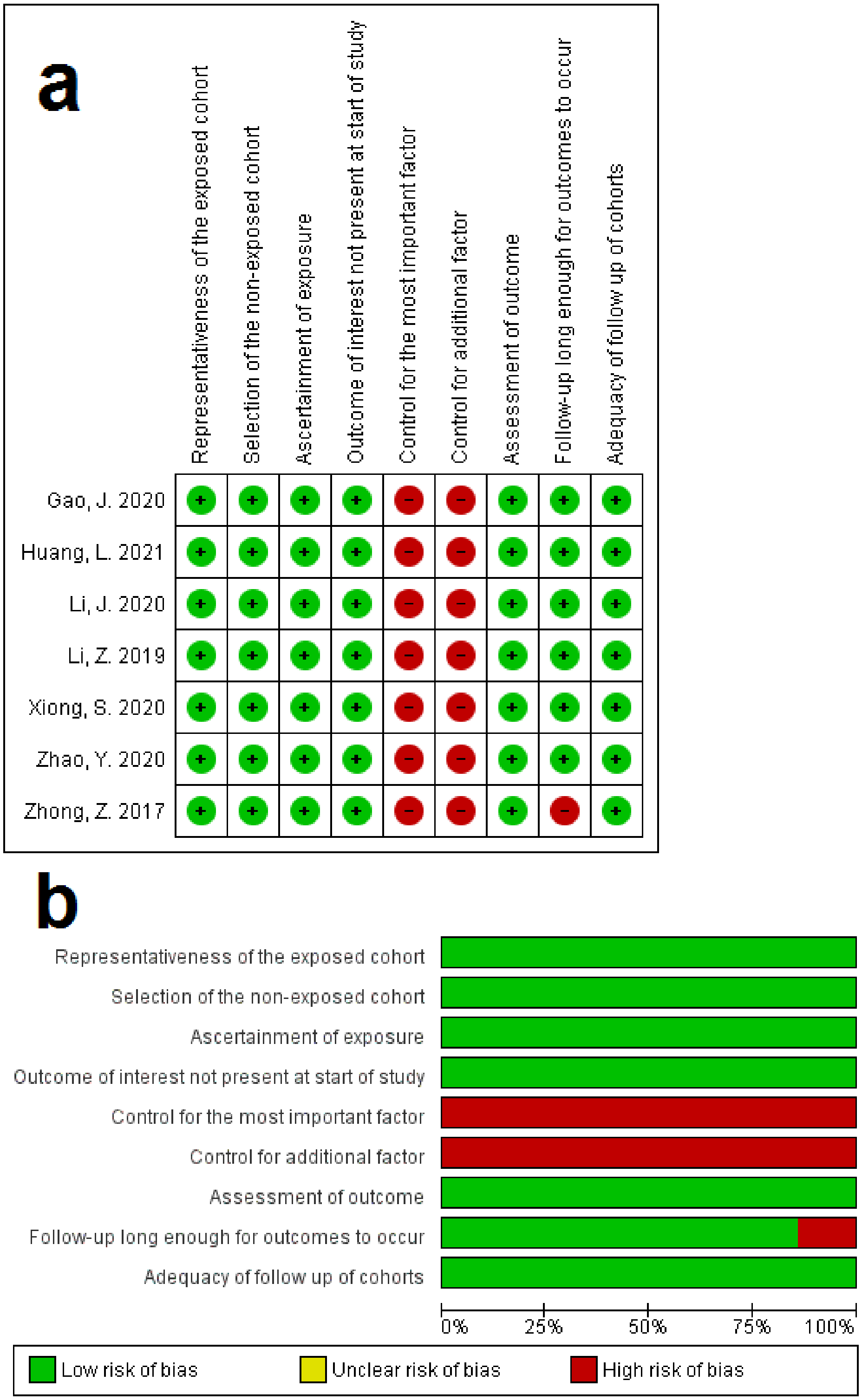

Figure S1 Quality assessment of the included studies in prognostic analysis using the Newcastle-Ottawa scale regarding (a) each included study, and (b) each risk of bias item

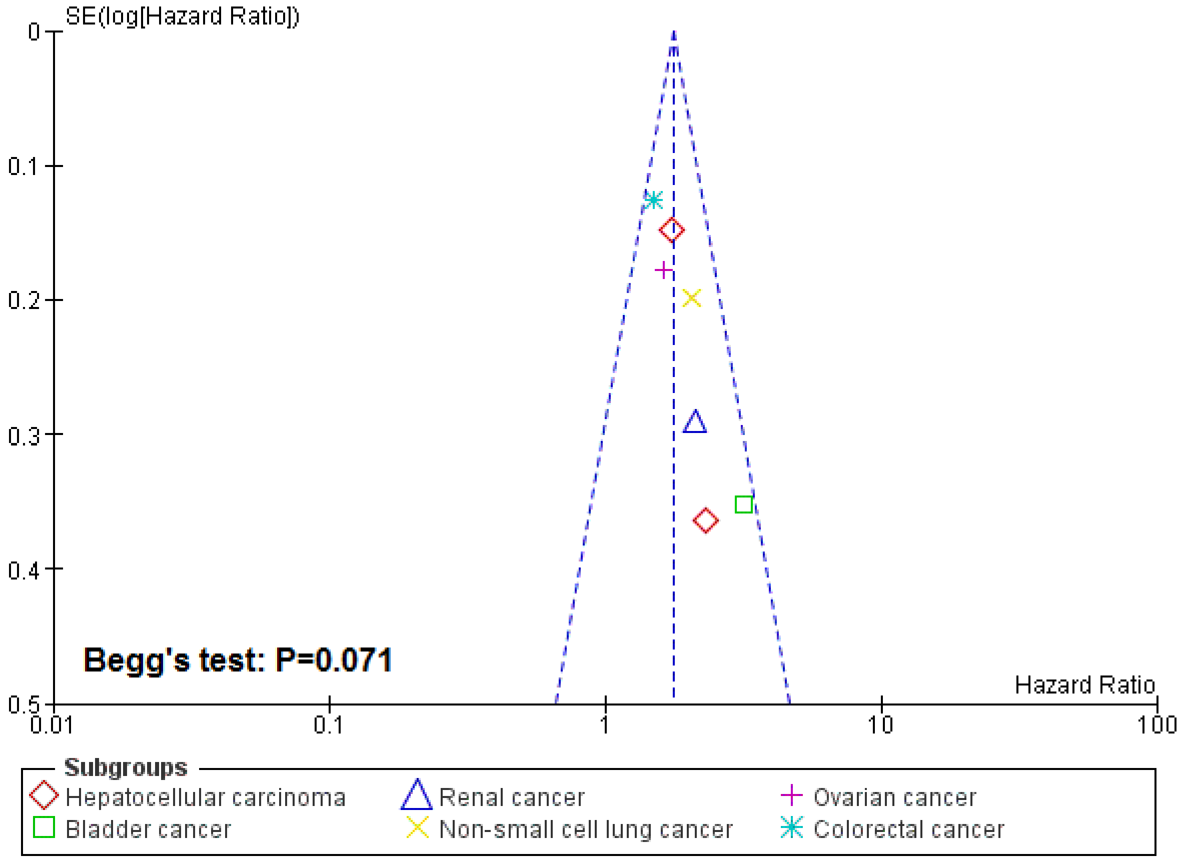

Figure S2 Funnel plot and Begg's test for assessment of publication bias in prognostic meta-analysis

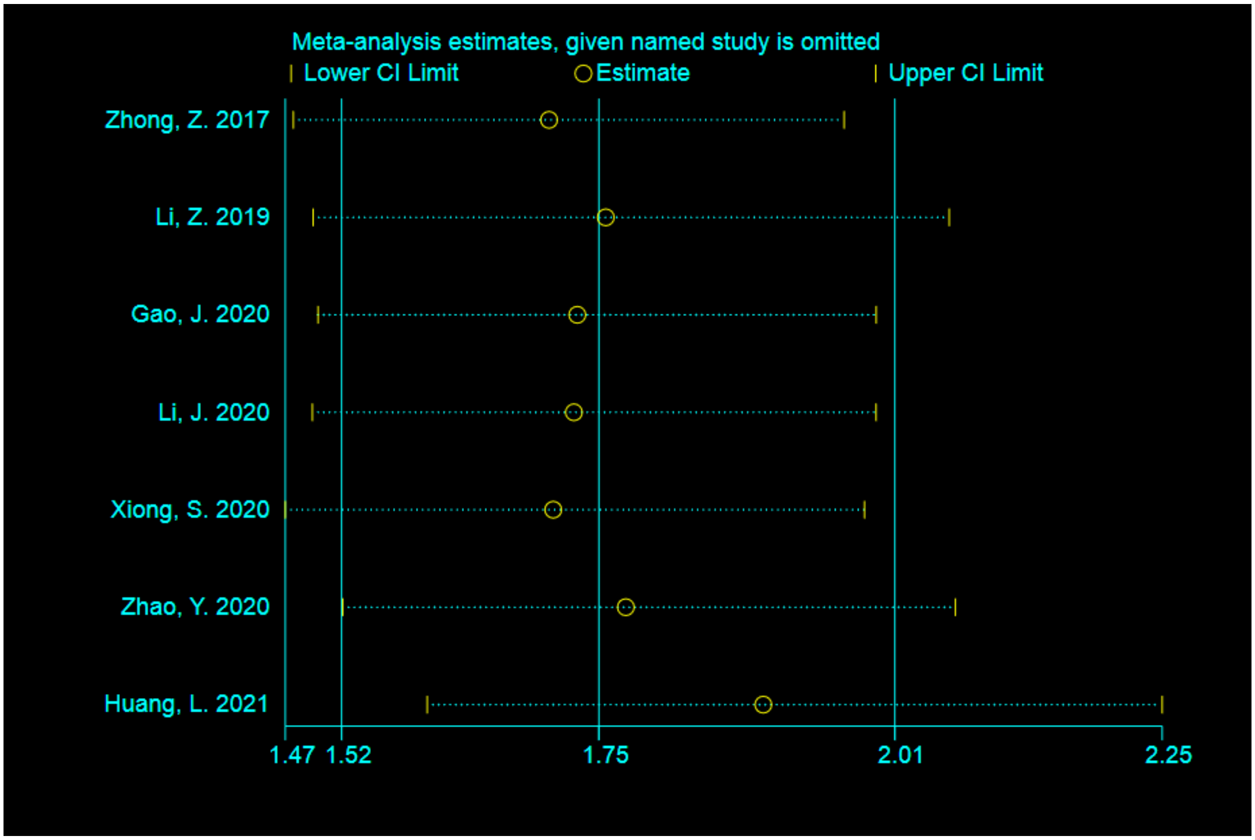

Figure S3 Sensitivity analysis for the prognostic meta-analysis
